# INHIBITION OF AUTOPHAGY RESTORES IMMUNOMODULATORY CAPACITY OF SENESCENT MSCS VIA COX-2/PGE2 PATHWAY

**DOI:** 10.1093/geroni/igad104.3434

**Published:** 2023-12-21

**Authors:** Yang Ma

**Affiliations:** Zhejiang university School of Medicine, Hangzhou, Zhejiang, China (People’s Republic)

## Abstract

Mesenchymal stem cells (MSCs) have been increasingly utilized in many therapeutic applications including autoimmune diseases and tissue repair due to their regenerative and immunomodulatory capacity. In vitro expansion will cause cell senescence and compromise their therapeutic effect. Therefore, underlying mechanisms of MSCs senescence during in vitro culture and methods of enhancing their therapeutic capacity need to be explored. Autophagy is a key regulator of biological properties of MSCs and is highly related to aging process. Whether manipulation of autophagy could restore immunomodulatory capacity of senescent MSCs after in vitro culture is still unclear. We demonstrated here that autophagy was significantly increased in aged MSCs compared with young MSCs. Inhibition of autophagy by BafA and CQ enhanced their immunomodulatory capacity of eliciting M2 polarization of macrophages. COX-2/PGE2 pathway was activated by inhibition of autophagy in this process. Inhibition of COX-2/PGE2 pathway of aged MSCs by SC-236 abandoned the enhanced immunomodulatory capacity of aged MSCs. Inhibition of autophagy by CQ of aged MSCs also increased their therapeutic capacity in cutaneous wound models of rats by increasing their capacity of eliciting M2 polarization of macrophages. Taken together, this work indicates that inhibition of autophagy of MSCs after in vitro expansion could ameliorate senescence-related changes in MSCs and enhance their immunomodulatory capacity. This may represent a potential therapeutic avenue to clinical application of MSCs.

